# The preoperative platelet to neutrophil ratio and lymphocyte to monocyte ratio are superior prognostic indicators compared with other inflammatory biomarkers in ovarian cancer

**DOI:** 10.3389/fimmu.2023.1177403

**Published:** 2023-06-30

**Authors:** Qian Song, Song-Xiao Xu, Jun-Zhou Wu, Lin Ling, Sheng Wang, Xin-Hua Shu, Dan-Ni Ying, Wang-Wei Pei, Yu-Chen Wu, Su-Fang Sun, Yi-Ning Zhang, Si-Hang Zhou, Zhu-Yan Shao

**Affiliations:** ^1^ Department of Clinical Laboratory, Zhejiang Cancer Hospital, Hangzhou Institute of Medicine (HIM), Chinese Academy of Sciences, Hangzhou, Zhejiang, China; ^2^ Cancer Research Institute, Zhejiang Cancer Hospital, Hangzhou Institute of Medicine (HIM), Chinese Academy of Sciences, Hangzhou, Zhejiang, China; ^3^ Department of Gynaecology, Haining People’s Hospital, Haining, Zhejiang, China; ^4^ Department of Gynecological Oncology, Zhejiang Cancer Hospital, The Key Laboratory of Zhejiang Province for Aptamers and Theranostics, Hangzhou Institute of Medicine (HIM), Chinese Academy of Sciences, Hangzhou, Zhejiang, China

**Keywords:** ovarian cancer, neutrophil to lymphocyte ratio, lymphocyte to monocyte ratio, platelet to lymphocyte ratio, systemic immune-inflammation index, platelet to neutrophil ratio, prognosis

## Abstract

**Background:**

Previous studies have suggested that the ratios of immune-inflammatory cells could serve as prognostic indicators in ovarian cancer. However, which of these is the superior prognostic indicator in ovarian cancer remains unknown. In addition, studies on the prognostic value of the platelet to neutrophil ratio (PNR) in ovarian cancer are still limited.

**Methods:**

A cohort of 991 ovarian cancer patients was analyzed in the present study. Receiver operator characteristic (ROC) curves were utilized to choose the optimal cut-off values of inflammatory biomarkers such as neutrophil to lymphocyte ratio (NLR), lymphocyte to monocyte ratio (LMR), platelet to lymphocyte ratio (PLR), systemic immune-inflammation index (SII), and PNR. The correlation of inflammatory biomarkers with overall survival (OS) and relapse-free survival (RFS) was investigated by Kaplan-Meier methods and log-rank test, followed by Cox regression analyses.

**Results:**

Kaplan-Meier curves suggested that LMR<3.39, PLR≥181.46, and PNR≥49.20 had obvious associations with worse RFS (P<0.001, P=0.018, P<0.001). Multivariate analysis suggested that LMR (≥3.39 vs. <3.39) (P=0.042, HR=0.810, 95% CI=0.661-0.992) and PNR (≥49.20 vs. <49.20) (P=0.004, HR=1.351, 95% CI=1.103-1.656) were independent prognostic indicators of poor RFS. In addition, Kaplan-Meier curves indicated that PLR≥182.23 was significantly correlated with worse OS (P=0.039).

**Conclusion:**

Taken together, PNR and LMR are superior prognostic indicators compared with NLR, PLR, and SII in patients with ovarian cancer.

## 1 Introduction

Ovarian cancer is the most lethal gynecological malignancy, with an estimated 313,959 new cases and 207,252 deaths worldwide in 2020 (1). In early-stage ovarian cancer, complete surgical staging plays an important role in the selection of adjunctive therapies. Due to asymptomatic rapid progression, most ovarian cancer patients were newly diagnosed at advanced stages (2). The standard primary treatment for advanced stage ovarian cancer is cytoreductive surgery followed by platinum and taxane based chemotherapy (3). Ovarian cancer remains fatal due to its advanced stage at the time of diagnosis, resistance to chemotherapy and high relapse rate (4). According to the previous reports, the median OS and recurrence-free survival was 33.9 and 10.7 months in the surgery group, respectively (2). Therefore, there is an urgent need to evaluate potential prognostic indicators to guide therapeutic strategy, monitor treatment response, and identify patients at high risk of recurrence and death.

Accumulating studies suggest that circulating immune-inflammatory cells, including neutrophils (5), lymphocytes (6), monocytes (7), and platelets (8), play an important role in tumor progression. In addition, increasing numbers of reports have indicated that circulating immune-inflammatory cells in the peripheral blood may serve as novel prognostic indicators in various cancer, such as esophageal cancer, lung cancer, and hepatocellular cancer (9–13). Moreover, the ratios of immune-inflammatory cells such as NLR, LMR, PLR, and SII have been reported to be closely related to survival in ovarian cancer patients (14–17). However, which of these is the superior prognostic indicator in ovarian cancer remains unknown. Moreover, few studies have focused on the prognostic value of PNR in patients with ovarian cancer, although there was one study reported the association between PNR and the prognosis of ovarian cancer (18).

Therefore, we evaluated the prognostic values of NLR, LMR, PLR, SII, and PNR in ovarian cancer, and compared their capacity to predict survival in the present study.

## 2 Materials and methods

### 2.1 Patient selection

A cohort of ovarian cancer patients who received surgical treatment at Zhejiang Cancer Hospital from August 2006 to October 2018 was retrospectively analyzed. Patients who underwent neoadjuvant treatment, those without complete clinical data, and those with previous or concomitant other cancers were excluded. Finally, a total of 991 ovarian cancer patients were included in this retrospective study. Clinical data, including clinical features and laboratory data, were obtained from the electronic medical record system. The laboratory data, including CA125 levels and blood routines tests, were collected within one week before surgery. Platelet, neutrophil, lymphocyte, and monocyte counts were examined by a blood routine test. NLR was calculated as neutrophil count/lymphocyte count. LMR was calculated as lymphocyte count/monocyte count. PLR was calculated as platelet count/lymphocyte count. SII was calculated as (platelet count × neutrophil count)/lymphocyte count. PNR was calculated as platelet count/neutrophil count. Ovarian cancer stage was classified based on the International Federation of Gynecology and Obstetrics (FIGO).

### 2.2 Statistical analysis

Continuous variables, which do not conform to the normal distribution, were presented as median and interquartile range and compared using non-parametric tests. Categorical variables were shown as absolute values and analyzed by the chi-square test. Differences in OS and RFS were compared using the log-rank tests. The curves of OS and RFS were plotted by the Kaplan-Meier method using the GraphPad Prism 7 software. Univariate analysis was utilized to investigate the association between the prognostic indicators and survival. Cox regression analysis was used to evaluate whether prognostic indicators were significant independent factors. We chose the optimal cut-off values for NLR, LMR, PLR, SII, and PNR using the ROC curves. Statistical analysis was carried out using SPSS software version 19.0 (SPSS, Chicago, IL, USA). A P value less than 0.05 was considered statistically significant.

## 3 Results

### 3.1 Optimal cut-off value of inflammatory biomarkers

**Figure 1:**
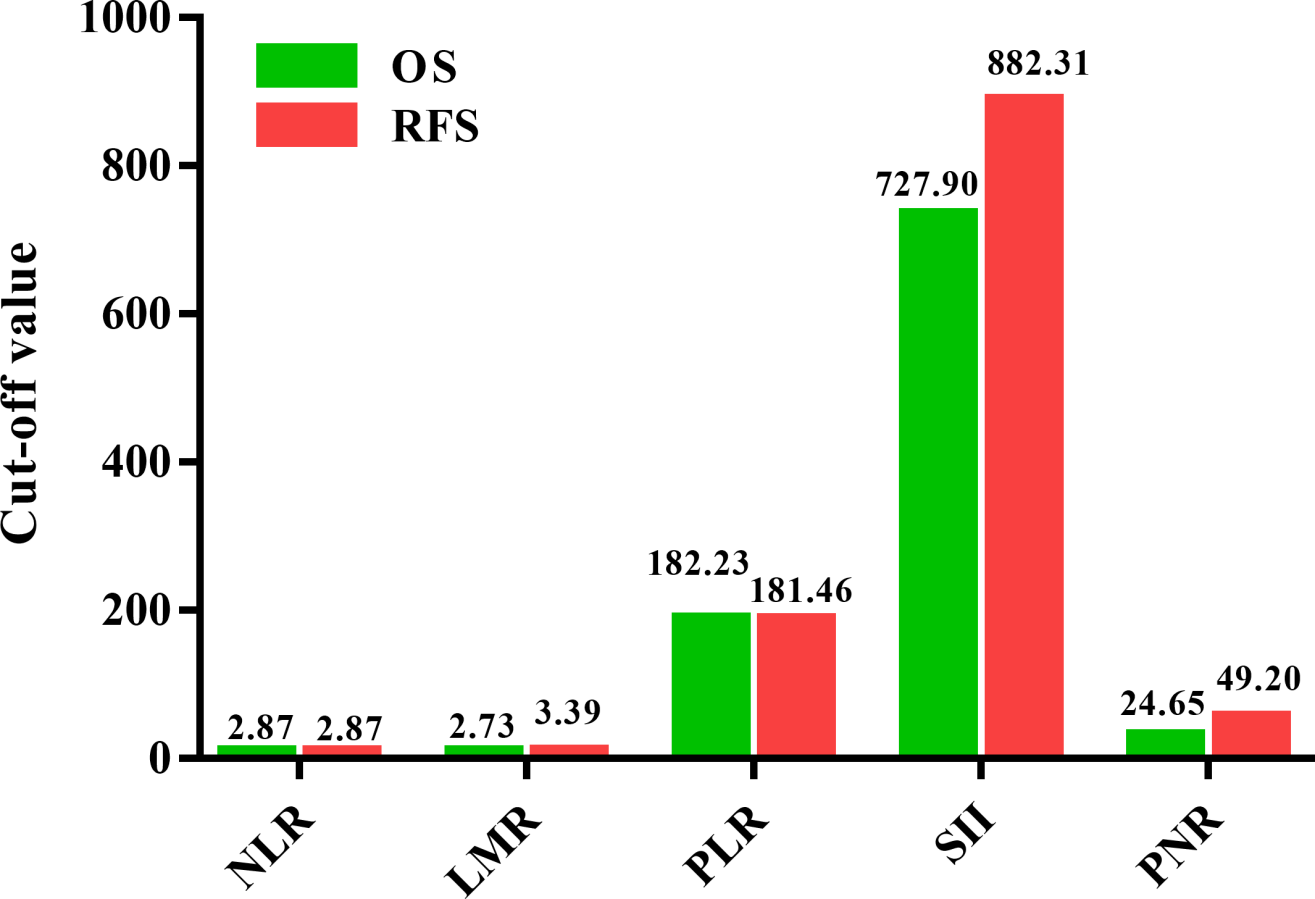
Comparison of the cut-off values for inflammatory biomarkers on overall survival (OS) and relapse-free survival (RFS) in patients with ovarian cancer.

The ROC curves were utilized to choose the optimal cut-off values of NLR, LMR, PLR, SII, and PNR for predicting OS and RFS. Our results indicated that the optimal cut-off values of NLR, LMR, PLR, SII, and PNR for predicting OS were 2.87, 2.73, 182.23, 727.90, and 24.65 (**Figure 1**). In addition, the optimal cut-off values of NLR, LMR, PLR, SII, and PNR to indicate RFS were 2.87, 3.39, 181.46, 882.31, and 49.20, respectively (**Figure 1**). Due to differences between optimal cut-off values of OS and those of RFS, the cut-off values of OS and RFS were both utilized for further analysis. Patients were divided into two groups (NLR<2.87 vs. ≥2.87; LMR<2.73 vs. ≥2.73; PLR<182.23 vs. ≥182.23; SII<727.90 vs.≥727.90; PNR<24.65 vs. ≥24.65) for OS analysis. Moreover, we stratified patients into two groups (NLR<2.87 vs. ≥2.87; LMR<3.39 vs. ≥3.39; PLR<181.46 vs. ≥181.46; SII<882.31 vs.≥882.31; PNR<49.20 vs. ≥49.20) for RFS analysis.

### 3.2 Clinicopathological features and inflammatory biomarkers

The age ranged from 23 to 83 years, with a median age of 55 years among the 991 patients enrolled. 581 patients were menopause and 410 patients were non-menopause. There were 55 patients whose FIGO stage was I, 84 patients whose FIGO stage was II, 682 patients whose FIGO stage was III, and 170 patients whose FIGO stage was IV. 327 patients had a family history of cancer, 664 patients without family history of cancer. There were 859 patients had residual disease ≤ 1cm, 132 patients had residual disease >1cm. 829 patients were serous, 162 patients were other histology. There were 376 patients with well grade, 233 patients with moderate grade, 275 patients with poor grade, and 107 patients whose grade were unknown. 548 patients had lymph node metastasis, and 443 patients without lymph node metastasis. The median of CA125 at diagnosis was 846.45 and the range of CA125 was between 270.48 and 2062.10. There were only 51 patients within the reference range (0-35 U/ml), 940 patients beyond the reference range (>35 U/ml) (**Table 1**). The quartile interval of NLR, LMR, PLR, SII, and PNR were 2.15-4.43, 2.17-4.21, 128.67-267.04, 489.19-1419.68, and 30.61-64.44. The medians of NLR, LMR, PLR, SII, and PNR were 3.05, 3.00, 182.49, 797.29, and 43.61. Details of clinicopathological features and inflammatory biomarkers were shown in **Table 1**.

**Table 1:** Baseline Characteristics of the ovarian cancer patients enrolled in the study.

Charateristics	Total (N = 991)
**Age[median (range),years]**	55 (23-83)
≤55	521
>55	470
Menopause	
Yes	581
No	410
FIGO stage	
I	55
II	84
III	682
IV	170
Family history of cancer	
Yes	327
No	664
Residual disease	
≤1cm	859
>1cm	132
Histology	
Serous	829
Other	162
Grade	
Well	376
Moderate	233
Poor	275
Unknown	107
Lymph node status	
Positive	548
Negative	443
**CA125 at diagnosis**	846.45 (270.48-2062.10)
≤35 U/ml	51
>35 U/ml	940
**Platelet (10^9^/L)**	267.00 (207.00-345.00)
**Neutrophil (10^9^/L)**	4.40 (3.30-5.73)
**Lymphocyte (10^9^/L)**	1.40 (1.10-1.80)
**Monocyte (10^9^/L)**	0.50 (0.40-0.60)
**NLR**	3.05 (2.15-4.43)
**LMR**	3.00 (2.17-4.21)
**PLR**	182.49 (128.67-267.04)
**SII**	797.29 (489.19-1419.68)
**PNR**	43.61 (30.61-64.44)

### 3.3 Prognostic value of inflammatory biomarkers

**Figure 2:**
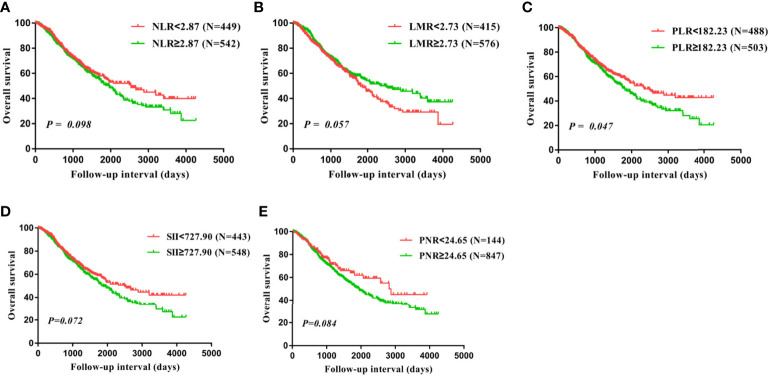
Overall survival (OS) analysis in all 991 patients with ovarian cancer based on the NLR **(A)**, LMR **(B)**, PLR **(C)**, SII **(D)**, and PNR **(E)**.

**Figure 3:**
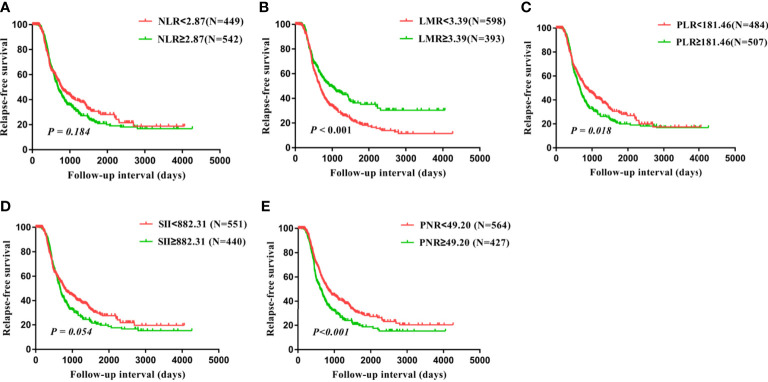
Relapse-free survival (RFS) analysis in all 991 patients with ovarian cancer based on the NLR **(A)**, LMR **(B)**, PLR **(C)**, SII **(D)**, and PNR **(E)**.

Univariate analysis and kaplan-Meier survival analysis were carried out to investigate the prognostic value of inflammatory biomarkers. Our findings suggested that PLR was significantly correlated with the prognosis of OS (P=0.047, **Table 2**; **Figure 2**). Subsequently, these factors were further assessed by Cox multivariate analysis, and the findings indicated that FIGO stage and residual disease had a significant correlation with OS, suggesting that FIGO stage and residual disease are independent prognostic indicators of OS. However, no significant relationship between inflammatory biomarkers and prognosis was observed using COX multivariate analysis (**Table 2**). In addition, LMR, PLR, and PNR were significantly related to prognosis of RFS based on the univariate analysis and kaplan-Meier survival analysis (P<0.001, P=0.018, P<0.001, **Table 3**; **Figure 3**). Moreover, the univariate analysis suggested that FIGO stage, residual disease, histology, and lymph node metastasis were significantly related to prognosis (all P<0.05, **Table 3**). Cox multivariate analysis showed that LMR (P=0.042, HR=0.810, 95% CI=0.661-0.992) and PNR (P=0.004, HR=1.351, 95% CI=1.103-1.656) were independent prognostic indicators of poor RFS (**Table 3**).

**Table 2:** Prognostic significance of inflammation parameters for the overall survival of surgically resectable ovarian cancer.

Variables	Univariate			Multivariate		
	HR	95% CI	*P* value	HR	95% CI	*P* value
**Age (>55 vs.≤55)**	1.242	1.008-1.530	**0.042**	0.956	0.686-1.332	0.791
**Menopause (No vs. Yes)**	1.336	1.079-1.655	**0.008**	1.373	0.979-1.925	0.066
FIGO stage						
I	0.426	0.210-0.867	**0.019**	0.449	0.219-0.917	**0.028**
II	0.528	0.317-0.880	**0.014**	0.573	0.343-0.959	**0.034**
III	1.074	0.796-1.450	0.640	1.029	0.760-1.392	0.856
IV	1.000			1.000		
**Family history of cancer (No vs. Yes)**	0.909	0.727-1.137	0.404			
**Residual disease (>1cm vs. ≤1cm)**	1.584	1.224-2.050	**<0.001**	1.386	1.066-1.802	**0.015**
**Histology (Other vs. Serous)**	1.113	0.846-1.465	0.443			
Grade						
Well	0.862	0.627-1.185	0.360			
Moderate	1.088	0.848-1.396	0.506			
Poor	1.000					
**Lymph node status (Negative vs. Positive)**	1.184	0.959-1.462	0.116			
**CA125 at diagnosis (>35 U/ml vs.≤35 U/ml)**	1.389	0.781-2.470	0.264			
**NLR (≥2.87 vs.<2.87)**	1.195	0.968-1.476	0.098			
**LMR (≥2.73 vs.<2.73)**	0.817	0.663-1.006	0.057			
**PLR (≥182.23 vs.<182.23)**	1.235	1.002-1.522	**0.047**	1.158	0.935-1.434	0.179
**SII (≥727.90 vs.<727.90)**	1.214	0.983-1.499	0.072			
**PNR(≥24.65 vs.<24.65)**	1.320	0.963-1.810	0.085			

The bold values represent P<0.05.

**Table 3:** Prognostic significance of inflammation parameters for the relapse-free survival of surgically resectable ovarian cancer.

Variables	Univariate			Multivariate		
	HR	95% CI	*P* value	HR	95% CI	*P* value
**Age (>55 vs.≤55)**	1.037	0.870-1.236	0.687			
**Menopause (No vs. Yes)**	1.046	0.878-1.247	0.615			
FIGO stage						
I	0.084	0.034-0.207	**<0.001**	0.116	0.046-0.290	**<0.001**
II	0.217	0.131-0.359	**<0.001**	0.273	0.161-0.461	**<0.001**
III	0.787	0.631-0.981	**0.033**	0.812	0.649-1.016	0.069
IV	1.000			1.000		
**Family history of cancer (No vs. Yes)**	0.871	0.722-1.051	0.149			
**Residual disease (>1cm vs. ≤1cm)**	1.599	1.257-2.035	**<0.001**	1.349	1.058-1.720	**0.016**
**Histology (Other vs. Serous)**	0.473	0.356-0.630	**<0.001**	0.647	0.484-0.865	**0.003**
Grade						
Well	1.151	0.917-1.445	0.226			
Moderate	1.202	0.962-1.502	0.106			
Poor	1.000					
**Lymph node status (Negative vs. Positive)**	1.584	1.324-1.896	**<0.001**	1.063	0.882-1.283	0.521
**CA125 at diagnosis (>35 U/ml vs.≤35 U/ml)**	0.949	0.629-1.430	0.801			
**NLR (≥2.87 vs.<2.87)**	1.127	0.945-1.344	0.184			
**LMR (≥3.39 vs.<3.39)**	0.685	0.568-0.827	**<0.001**	0.810	0.661-0.992	**0.042**
**PLR (≥181.46 vs.<181.46)**	1.235	1.036-1.472	**0.018**	0.890	0.718-1.104	0.288
**SII (≥882.31 vs.<882.31)**	1.187	0.997-1.415	0.055			
**PNR(≥49.20 vs.<49.20)**	1.402	1.177-1.670	**<0.001**	1.351	1.103-1.656	**0.004**

The bold values represent P<0.05.

### 3.4 Correlation of PNR, LMR and patient characteristics

Due to the significant relationships between LMR, PNR, and prognosis, the clinical features of patients grouped by LMR and PNR were adopted and shown in **Tables 4**, **5**. Our findings suggested that LMR had a significant close correlation with FIGO stage, histology, lymph node status, CA125 at diagnosis, platelet, neutrophil, lymphocyte, monocyte, NLR, LMR, PLR, SII, and PNR (all P<0.05, **Table 4**). PNR was significantly correlated with age, menopause, FIGO stage, grade, CA125 at diagnosis, platelet, neutrophil, lymphocyte, monocyte, NLR, LMR, PLR, SII, and PNR (all P<0.05, **Table 5**). Because of no obvious correlation between NLR, PLR, SII, and prognosis, the characteristics of patients divided by NLR, PLR, and SII were not presented.

**Table 4:** Relationship between LMR and clinicopathological features in patients with ovarian cancer.

Charateristics	LMR<3.39 (N=598)	LMR≥3.39 (N=393)	*P* value
**Age[median (range),years]**	54 (35-81)	56 (23-83)	0.052
≤55	328	193	0.077
>55	270	200	
Menopause			
Yes	339	242	0.126
No	259	151	
FIGO stage			
I	20	35	**<0.001**
II	40	44	
III	430	252	
IV	108	62	
Family history of cancer			
Yes	209	118	0.107
No	389	275	
Residual disease			
≤1cm	509	350	0.074
>1cm	89	43	
Histology			
Serous	513	316	**0.025**
Other	85	77	
Grade			
Well	214	162	0.133
Moderate	148	85	
Poor	175	100	
Lymph node status			
Positive	350	198	**0.012**
Negative	248	195	
**CA125 at diagnosis**	1181.30 (386.90-2409.68)	571.75 (159.60-1381.98)	**<0.001**
≤35 U/ml	26	25	0.160
>35 U/ml	572	368	
**Platelet (10^9^/L)**	283.00 (211.00-370.75)	242.50 (196.75-315.00)	**<0.001**
**Neutrophil (10^9^/L)**	4.90 (3.63-6.38)	3.80 (2.80-4.73)	**<0.001**
**Lymphocyte (10^9^/L)**	1.30 (1.00-1.50)	1.70 (1.40-2.03)	**<0.001**
**Monocyte (10^9^/L)**	0.60 (0.40-0.70)	0.40 (0.30-0.50)	**<0.001**
**NLR**	3.83 (2.83-5.63)	2.22 (1.60-2.84)	**<0.001**
**LMR**	2.33 (1.75-2.80)	4.50 (4.00-5.50)	**<0.001**
**PLR**	233.06 (161.57-336.75)	143.01 (108.25-185.35)	**<0.001**
**SII**	1106.41 (680.39-1873.56)	530.29 (343.00-782.12)	**<0.001**
**PNR**	49.20 (32.14-71.23)	38.60 (27.99-55.27)	**<0.001**

The bold values represent P<0.05.

**Table 5:** Relationship between PNR and clinicopathological features in patients with ovarian cancer.

Charateristics	PNR<49.20 (N=564)	PNR≥49.20 (N=427)	*P* value
**Age[median (range),years]**	56 (23-83)	53 (33-80)	**0.003**
≤55	268	253	**<0.001**
>55	296	174	
Menopause			
Yes	351	230	**0.008**
No	213	197	
FIGO stage			
I	35	20	**0.020**
II	60	24	
III	379	303	
IV	90	80	
Family history of cancer			
Yes	186	141	0.989
No	378	286	
Residual disease			
≤1cm	487	372	0.723
>1cm	77	55	
Histology			
Serous	467	362	0.405
Other	97	65	
Grade			
Well	196	180	**0.033**
Moderate	145	88	
Poor	163	112	
Lymph node status			
Positive	304	244	0.309
Negative	260	183	
**CA125 at diagnosis**	684.85 (213.15-1759.40)	1105.35 (371.98-2372.88)	**<0.001**
≤35 U/ml	28	23	0.766
>35 U/ml	536	404	
**Platelet (10^9^/L)**	244.00 (193.00-312.50)	306.00 (226.75-387.00)	**<0.001**
**Neutrophil (10^9^/L)**	4.85 (3.83-6.30)	3.70 (2.70-4.90)	**<0.001**
**Lymphocyte (10^9^/L)**	1.70 (1.40-2.00)	1.20 (0.90-1.40)	**<0.001**
**Monocyte (10^9^/L)**	0.50 (0.40-0.70)	0.40 (0.30-0.50)	**<0.001**
**NLR**	2.94 (2.12-4.15)	3.27 (2.16-4.90)	**0.008**
**LMR**	3.31 (2.33-4.50)	2.71 (2.00-3.67)	**<0.001**
**PLR**	146.85 (109.77-190.76)	266.67 (188.54-372.32)	**<0.001**
**SII**	712.33 (458.94-1131.67)	944.76 (548.38-1788.06)	**<0.001**
**PNR**	31.99 (24.38-39.47)	68.24 (58.19-88.17)	**<0.001**

The bold values represent P<0.05.

### 3.5 Subgroup analysis based on other clinical features

**Figure 4:**
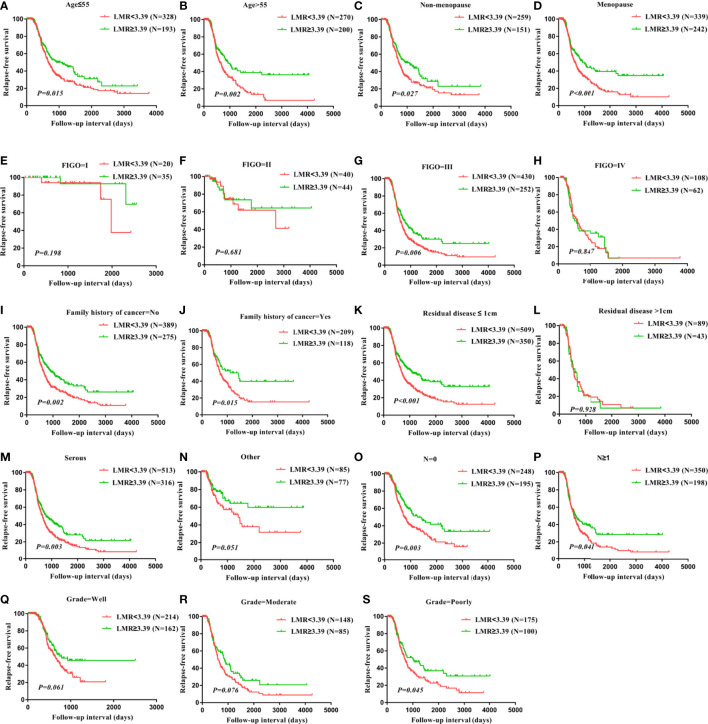
Correlation of LMR with relapse-free survival in all subtypes of ovarian cancer. RFS in patients with differential LMR level in patients with age ≤ 55 years **(A)**, patients with age>55 years **(B)**, patients with non-menopause **(C)**, patients with menopause **(D)**, patients with FIGO stage I **(E)**, patients with FIGO stage II **(F)**, patients with FIGO stage III **(G)**, patients with FIGO stage IV **(H)**, patients without family history of cancer **(I)**, patients with family history of cancer **(J)**, patients with residual disease ≤ 1cm **(K)**, patients with residual disease >1cm **(L)**, patients with histology serous **(M)**, patients with histology other **(N)**, patients without lymph node metastasis **(O)**, patients with lymph node metastasis **(P)**, patients with grade well **(Q)**, patients with grade moderate **(R)**, patients with grade poorly **(S)**.

To evaluate the subgroups of patients with ovarian cancer impacted by LMR and PNR, patients were divided based on age, menopause status, FIGO stage, a family history of cancer, residual disease, histology, lymph node status, and grade. RFS of age ≤ 55 patients (P=0.015), age>55 patients (P=0.002), patients without menopause (P=0.027), patients with menopause (P<0.001), patients with FIGO stage III (P=0.006), patients without a family history of cancer (P=0.002), patients with a family history of cancer (P=0.015), patients with residual disease ≤ 1 cm (P<0.001), patients with serous (P=0.003), patients without lymph node metastasis (P=0.003), patients with lymph node metastasis (P=0.041), patients with poor grade (P=0.045) were significantly worse for those with LMR<3.39, but RFS did not differ in patients whose FIGO stage was I, patients whose FIGO stage was II, patients whose FIGO stage was IV, patients with residual disease>1 cm, patients with other histology, patients with well grade, patients with moderate grade (**Figure 4**). Taken together, these findings suggest that LMR was significantly associated with clinical outcomes in ovarian cancer patients with FIGO stage III, with residual disease ≤ 1 cm, with serous, and with a poor grade. The status of age, menopause, a family history of cancer, and lymph node metastasis do not influence the significant relationship between LMR and survival in ovarian cancer patients.

**Figure 5:**
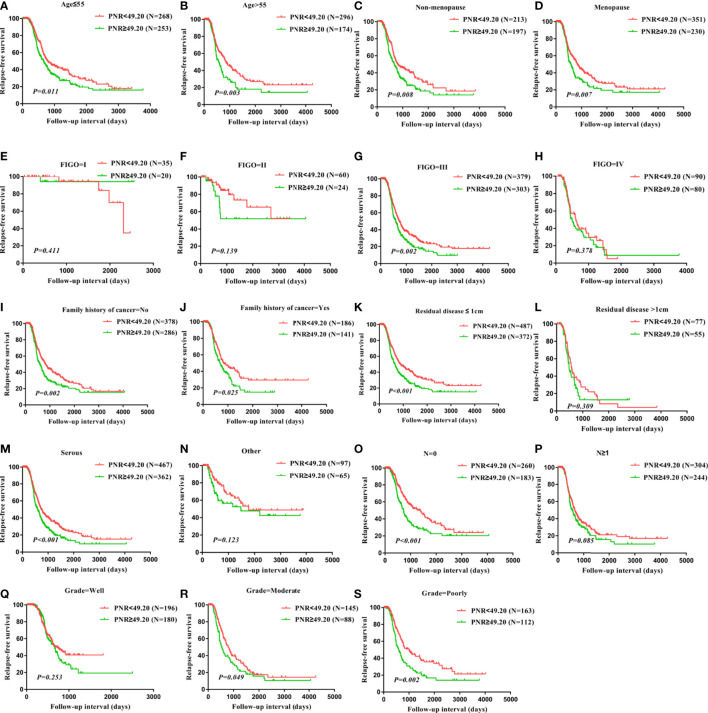
Correlation of PNR with relapse-free survival in all subtypes of ovarian cancer. RFS in patients with differential PNR level in patients with age ≤ 55 years **(A)**, patients with age>55 years **(B)**, patients with non-menopause **(C)**, patients with menopause **(D)**, patients with FIGO stage I **(E)**, patients with FIGO stage II **(F)**, patients with FIGO stage III **(G)**, patients with FIGO stage IV **(H)**, patients without family history of cancer **(I)**, patients with family history of cancer **(J)**, patients with residual disease ≤ 1cm **(K)**, patients with residual disease >1cm **(L)**, patients with histology serous **(M)**, patients with histology other **(N)**, patients without lymph node metastasis **(O)**, patients with lymph node metastasis **(P)**, patients with grade well **(Q)**, patients with grade moderate **(R)**, patients with grade poorly **(S)**.

In addition, RFS of age ≤ 55 patients (P=0.011), age>55 patients (P=0.003), patients without menopause (P=0.008), patients with menopause (P=0.007), patients with FIGO stage III (P=0.002), patients without a family history of cancer (P=0.002), patients with a family history of cancer (P=0.025), patients with residual disease ≤ 1cm (P<0.001), patients with serous (P<0.001), patients without lymph node metastasis (P<0.001), patients with moderate grade (P=0.049), patients with poor grade (P=0.002) were significantly worse for those with PNR≥49.20, but RFS did not differ in patients whose FIGO stage was I, patients whose FIGO stage was II, patients whose FIGO stage was IV, patients with residual disease>1 cm, patients with other histology, patients with well grade, patients with moderate grade (**Figure 5**). Together, these results indicate that PNR was related to survival in ovarian cancer patients with FIGO stage III, with residual disease ≤ 1cm, with serous, without lymph node metastasis, with moderate grade, and with poor grade. The status of age, menopause, and a family history of cancer do not affect the correlation between PNR and clinical outcomes in ovarian cancer patients.

## 4 Discussion

Numerous evidences have shown that inflammatory biomarkers have a close association with clinical features and survival in patients with ovarian cancer (10–15). Accordingly, our findings indicated that LMR was related to FIGO stage, histology, and lymph node status. In addition, PNR had a significant relationship with FIGO stage and grade. Kaplan-Meier curves and multivariate analysis both indicated that LMR and PNR were significantly correlated with recurrence free survival in patients with ovarian cancer.

However, the mechanisms of the relationship between inflammatory biomarkers and survival in patients with ovarian cancer remain unknown. There are several explanations. First, LMR is the calculated data between lymphocytes and monocytes. LMR that is associated with survival in patients with ovarian cancer is on behalf of lymphocytes and monocytes. Previous studies have shown that lymphocytes contribute to inhibiting tumor cell proliferation, migration, and invasion (19). Cytotoxic lymphocytes play an important role in eliminating residual tumor cells and being utilized in immunological therapy (20, 21). Monocytes have been proven to promote tumor development by differentiating to tumor-associated macrophages (TAMs). After receiving signals from tumor-derived chemokines, TAMs are recruited to the tumor site (22). Infiltration of TAMs has been reported to have a close correlation with the prognosis of various cancers (23, 24). Above all, this may partly explain why low LMR was related to worse recurrence free survival in patients with ovarian cancer. In addition, elevated PNR has a significant correlation with worse prognosis in patients with ovarian cancer. PNR is on behalf of platelets and neutrophils. Platelets have been proven to facilitate tumor development, while neutrophils have been shown to inhibit tumorigenesis (25, 26). Tumor-activated platelets play an important role in tumor progression by facilitating angiogenesis and metastasis (27). Moreover, platelets contribute to supervising tumor processes by regulating cancer cell recognition and effector functions that were mediated by the natural killer cells (27). Tumor associated neutrophils (TANs) play an important role in the tumor microenvironment by secreting chemokines and cytokines. N1 TANs, the subgroup of TANs, contribute to inhibiting the tumorigenesis through direct or indirect cytotoxicity (28). Tumor-entrained neutrophils (TENs) inhibit metastatic seeding in the lungs by generating H_2_O_2_. Thus, the inhibitory process mediated by neutrophils was induced at the metastatic site (29). This may partly explain why high PNR was related to poor recurrence free survival in patients with ovarian cancer. Second, low LMR and elevated PNR were significantly correlated with clinical features including FIGO stage, histology, stage, and lymph node status. Due to the association between these clinical features and the extent of tumor development, and consequently, affect the prognosis of patients with ovarian cancer. Third, systemic inflammatory response may affect the tolerance and compliance with active treatment (30), and thus influence prognosis in patients with ovarian cancer.

Clinical trials have shown that ovarian cancer patients receive little benefit from immunotherapy. The KEYNOTE-100 trial reported that an objective response rate (ORR) of 8% for the anti-programmed death 1 (PD-1) antibody pembrolizumab (31). In addition, another large immune therapy trial showed that the ORR for the anti-programmed death ligand 1 (PD-L1) antibody avelumab was 9.6% (32). Furthermore, the NINJA trial found that patients with platinum-resistant ovarian cancer who received nivolumab had no apparent improvement in OS and PFS compared to single-agent chemotherapy (33). These findings suggest that ovarian cancer patients receive little benefit from immunotherapy.

Up to now, there is no consistent conclusion about which inflammatory biomarker is the best prognostic indicator and the most clinically valuable in patients with ovarian cancer. A previous study has shown that PLR was superior to NLR as a predictor of OS and PFS in patients with ovarian cancer (34). A pilot study has indicated that PLR and monocyte-to-lymphocyte ratio (MLR) may serve as prognostic predictors compared with NLR (35). However, another study suggested that high NLR was related to survival in patients with ovarian cancer, while PLR and LMR were not associated with prognosis (36). In addition, few studies have focused on the prognostic value of SII and PNR in patients with ovarian cancer. A previous study has shown that high SII has a close relationship with poor prognosis in ovarian cancer patients (17). Elevated PNR was correlated with poor prognosis, in a cohort of 94 ovarian cancer patients in a pilot study (18). Our results indicated that LMR and PNR may serve as independent prognostic predictors in patients with ovarian cancer compared to other inflammatory biomarkers such as NLR, PLR, and SII. Although elevated PLR was associated with poor OS and RFS, PLR was not an independent prognostic indicator. NLR and SII were not significantly correlated with OS and RFS in the present study. These findings confirmed that LMR and PNR were superior compared with other inflammatory biomarkers for predicting survival among patients with ovarian cancer.

There is still controversy about which are the optimal cut-off values of these inflammatory biomarkers for predicting survival. Cut-off values were calculated by different methods in various studies (9, 15, 16, 35). To date, there is no standard method to establish a universal cut-off value. We used ROC curves to dichotomize the inflammatory biomarkers and chose the optimal cut-off value. Our results indicated that the optimal cut-off values of NLR, LMR, PLR, SII, and PNR for predicting OS were 2.87, 2.73, 182.23, 727.90, and 24.65. In addition, the optimal cut-off values of NLR, LMR, PLR, SII, and PNR for indicating RFS were 2.87, 3.39, 181.46, 882.31, and 49.20, respectively. However, the cut-off values identified in the present study may not be suitable for other studies. Therefore, these findings need to be verified in a multicenter.

Our study has some limitations: first, we did not explore the subgroup analysis based on the postoperative adjuvant treatments such as chemotherapy and targeted therapy, due to the lack of relevant data. Second, the present study was a retrospective design and single center. The prospective and multicenter study may reinforce the conclusion that PNR and LMR are superior prognostic indicators compared with NLR, PLR, and SII in patients with ovarian cancer.

## 5 Conclusion

Taken together, these findings indicate that PNR and LMR are superior prognostic indicators compared with NLR, PLR, and SII in patients with ovarian cancer. LMR<3.39 and PNR≥49.20 contributes to predicting relapse and assessing the patient risk stratification.

## Data availability statement

The raw data supporting the conclusions of this article will be made available by the authors, without undue reservation.

## Ethics statement

Written informed consent was obtained from the individual(s) for the publication of any potentially identifiable images or data included in this article.

## Author contributions

QS, S-XX and Z-YS conceived the project and wrote the manuscript. J-ZW, SW, LL, X-HS, D-NY, W-WP, Y-CW, S-FS, Y-NZ, and S-HZ managed data acquisition. QS, J-ZW, and SW participated in the data analysis. QS, and Z-YS participated in the discussion and language editing. S-XX reviewed the manuscript. All authors contributed to the article and approved the submitted version.

## References

[1] Sung H Ferlay J Siegel RL Laversanne M Soerjomataram I Jemal A. Global cancer statistics 2020: GLOBOCAN estimates of incidence and mortality worldwide for 36 cancers in 185 countries. CA Cancer J Clin (2021) 71:209–49. doi: 10.3322/caac.21660 33538338

[2] van Driel WJ Koole SN Sonke GS. Hyperthermic intraperitoneal chemotherapy in ovarian cancer. N Engl J Med (2018) 378:1363–4. doi: 10.1056/NEJMc1802033 29617590

[3] Binju M Padilla MA Singomat T Kaur P Suryo Rahmanto Y Cohen PA. Mechanisms underlying acquired platinum resistance in high grade serous ovarian cancer - a mini review. Biochim Biophys Acta Gen Subj. (2019) 1863:371–8. doi: 10.1016/j.bbagen.2018.11.005 30423357

[4] Bast RC Jr. Hennessy B Mills GB. The biology of ovarian cancer: new opportunities for translation. Nat Rev Cancer. (2009) 9:415–28. doi: 10.1038/nrc2644 PMC2814299 19461667

[5] Swierczak A Mouchemore KA Hamilton JA Anderson RL. Neutrophils: important contributors to tumor progression and metastasis. Cancer Metastasis Rev (2015) 34:735–51. doi: 10.1007/s10555-015-9594-9 26361774

[6] Yang F Wei Y Cai Z Yu L Jiang L Zhang C. Activated cytotoxic lymphocytes promote tumor progression by increasing the ability of 3LL tumor cells to mediate MDSC chemoattraction via fas signaling. Cell Mol Immunol (2015) 12:66–76. doi: 10.1038/cmi.2014.21 24769795 PMC4654365

[7] Delprat V Michiels C. A bi-directional dialog between vascular cells and monocytes/macrophages regulates tumor progression. Cancer Metastasis Rev (2021) 40:477–500. doi: 10.1007/s10555-021-09958-2 33783686 PMC8213675

[8] Sharma D Brummel-Ziedins KE Bouchard BA Holmes CE. Platelets in tumor progression: a host factor that offers multiple potential targets in the treatment of cancer. J Cell Physiol (2014) 229:1005–15. doi: 10.1002/jcp.24539 24374897

[9] Song Q Wu J Wang S Xu S. Perioperative change in neutrophil count predicts worse survival in esophageal squamous cell carcinoma. Future Oncol (2021) 17:4721–31. doi: 10.2217/fon-2021-0371 34431321

[10] Song Q Wu JZ Wang S. Perioperative change in lymphocyte count and prognosis in esophageal squamous cell carcinoma. J Thorac Dis (2019) 11:2332–9. doi: 10.21037/jtd.2019.06.02 PMC6626812 31372270

[11] Song Q Wu JZ Wang S. Postoperative monocyte count change is a better predictor of survival than preoperative monocyte count in esophageal squamous cell carcinoma. BioMed Res Int (2019) 2019:2702719. doi: 10.1155/2019/2702719 31485440 PMC6710746

[12] Yang W Yao Y Chen Y Jin F Zheng T Ai X. Dynamic changes of platelets before and after surgery predict the prognosis of patients with operable non-small cell lung cancer. J Cancer. (2022) 13:823–30. doi: 10.7150/jca.65129 PMC8824881 35154451

[13] Pang Q Liu C Qu K Liu S Berasain C. Conflicting relationship between platelets and prognosis of hepatocellular carcinoma: is platelet-derived serotonin involved in? Liver Int (2015) 35:2484. doi: 10.1111/liv.12843 25858667

[14] Yilmaz E Coskun EI Sahin N Ciplak B Ekici KMPV. NLR, and platelet count: new hematologic markers in diagnosis of malignant ovarian tumor. Eur J Gynaecol Oncol (2017) 38:346–9. 29693870

[15] Tang Y Hu HQ Tang YL Tang FX Zheng XM Deng LH. Preoperative LMR and serum CA125 level as risk factors for advanced stage of ovarian cancer. J Cancer. (2021) 12:5923–8. doi: 10.7150/jca.62090 PMC8408113 34476006

[16] Miao Y Yan Q Li S Li B Feng Y. Neutrophil to lymphocyte ratio and platelet to lymphocyte ratio are predictive of chemotherapeutic response and prognosis in epithelial ovarian cancer patients treated with platinum-based chemotherapy. Cancer biomark (2016) 17:33–40. doi: 10.3233/CBM-160614 27314290 PMC13020469

[17] Nie D Gong H Mao X Li Z. Systemic immune-inflammation index predicts prognosis in patients with epithelial ovarian cancer: a retrospective study. Gynecol Oncol (2019) 152:259–64. doi: 10.1016/j.ygyno.2018.11.034 30558974

[18] Bednarska K Krol E Glowacka E Romanowicz H Szyllo K Klink M. Analysis of preoperative blood platelet parameters in terms of diversity of epithelial ovarian cancer. Med (Baltimore). (2018) 97:e0180. doi: 10.1097/MD.0000000000010180 PMC5895358 29561432

[19] Bastid J Bonnefoy N Eliaou JF Bensussan A. Lymphocyte-derived interleukin-17A adds another brick in the wall of inflammation-induced breast carcinogenesis. Oncoimmunology (2014) 3:e28273. doi: 10.4161/onci.28273 25050201 PMC4063083

[20] Nazir T Islam A Omer MO Mustafa M. Lymphocytopenia; induced by vinorelbine, doxorubicin and cisplatin in human cancer patients. Breast Dis (2015) 35:1–4. doi: 10.3233/BD-140386 25171214

[21] Martinez-Lostao L Anel A Pardo J. How do cytotoxic lymphocytes kill cancer cells? Clin Cancer Res (2015) 21:5047–56. doi: 10.1158/1078-0432.CCR-15-0685 26567364

[22] Chanmee T Ontong P Konno K Itano N. Tumor-associated macrophages as major players in the tumor microenvironment. Cancers (Basel). (2014) 6:1670–90. doi: 10.3390/cancers6031670 PMC4190561 25125485

[23] Yang M Li Z Ren M Li S Zhang L Zhang X. Stromal infiltration of tumor-associated macrophages conferring poor prognosis of patients with basal-like breast carcinoma. J Cancer. (2018) 9:2308–16. doi: 10.7150/jca.25155 PMC6036715 30026826

[24] Zhang WJ Wang XH Gao ST Chen C Xu XY Sun Q. Tumor-associated macrophages correlate with phenomenon of epithelial-mesenchymal transition and contribute to poor prognosis in triple-negative breast cancer patients. J Surg Res (2018) 222:93–101. doi: 10.1016/j.jss.2017.09.035 29273380

[25] Bambace NM Holmes CE. The platelet contribution to cancer progression. J Thromb Haemost. (2011) 9:237–49. doi: 10.1111/j.1538-7836.2010.04131.x 21040448

[26] Garcia-Mendoza MG Inman DR Ponik SM Jeffery JJ Sheerar DS Van Doorn RR. Neutrophils drive accelerated tumor progression in the collagen-dense mammary tumor microenvironment. Breast Cancer Res (2016) 18:49. doi: 10.1186/s13058-016-0703-7 27169366 PMC4864897

[27] Stoiber D Assinger A. Platelet-leukocyte interplay in cancer development and progression. Cells (2020) 9:855–71. doi: 10.3390/cells9040855 PMC7226828 32244723

[28] Masucci MT Minopoli M Carriero MV. Tumor associated neutrophils. their role in tumorigenesis, metastasis, prognosis and therapy. Front Oncol (2019) 9:1146. doi: 10.3389/fonc.2019.01146 31799175 PMC6874146

[29] Granot Z Henke E Comen EA King TA Norton L Benezra R. Tumor entrained neutrophils inhibit seeding in the premetastatic lung. Cancer Cell (2011) 20:300–14. doi: 10.1016/j.ccr.2011.08.012 PMC3172582 21907922

[30] Scott HR McMillan DC Forrest LM Brown DJ McArdle CS Milroy R. The systemic inflammatory response, weight loss, performance status and survival in patients with inoperable non-small cell lung cancer. Br J Cancer. (2002) 87:264–7. doi: 10.1038/sj.bjc.6600466 PMC2364225 12177792

[31] Matulonis UA Shapira-Frommer R Santin AD Lisyanskaya AS Pignata S Vergote I. Antitumor activity and safety of pembrolizumab in patients with advanced recurrent ovarian cancer: results from the phase II KEYNOTE-100 study. Ann Oncol (2019) 30:1080–7. doi: 10.1093/annonc/mdz135 31046082

[32] Disis ML Taylor MH Kelly K Beck JT Gordon M Moore KM. Efficacy and safety of avelumab for patients with recurrent or refractory ovarian cancer: phase 1b results from the JAVELIN solid tumor trial. JAMA Oncol (2019) 5:393–401. doi: 10.1001/jamaoncol.2018.6258 30676622 PMC6439837

[33] Hamanishi J Takeshima N Katsumata N Ushijima K Kimura T Takeuchi S. Nivolumab versus gemcitabine or pegylated liposomal doxorubicin for patients with platinum-resistant ovarian cancer: open-label, randomized trial in Japan (NINJA). J Clin Oncol (2021) 39:3671–81. doi: 10.1200/JCO.21.00334 PMC8601279 34473544

[34] Zhang WW Liu KJ Hu GL Liang WJ. Preoperative platelet/lymphocyte ratio is a superior prognostic factor compared to other systemic inflammatory response markers in ovarian cancer patients. Tumour Biol (2015) 36:8831–7. doi: 10.1007/s13277-015-3533-9 26063409

[35] Sastra WIG Aditya PPK Gradiyanto OE Ketut S. Predictive value of preoperative inflammatory markers and serum CA 125 level for surgical outcome in Indonesian women with epithelial ovarian cancer. Cancer biomark (2022) 34:123–9. doi: 10.3233/CBM-201415 PMC12364174 34806598

[36] Li Z Hong N Robertson M Wang C Jiang G. Preoperative red cell distribution width and neutrophil-to-lymphocyte ratio predict survival in patients with epithelial ovarian cancer. Sci Rep (2017) 7:43001. doi: 10.1038/srep43001 28223716 PMC5320446

